# Early Onset of Severe Interstitial Pneumonitis Associated With Anti-PD-1 Immune Checkpoint Antibody After Pleurodesis

**DOI:** 10.7759/cureus.58798

**Published:** 2024-04-23

**Authors:** Sanshiro Haga, Akimasa Sekine, Eri Hagiwara, Taichi Kaneko, Takashi Ogura

**Affiliations:** 1 Department of Respiratory Medicine, Kanagawa Cardiovascular and Respiratory Center, Yokohama, JPN

**Keywords:** pneumonitis, pembrolizumab, ok-432, minocycline, pleurodesis

## Abstract

We present a case of lung adenocarcinoma with malignant pleural effusion. Nineteen days after pleurodesis using minocycline and OK-432 (picibanil), pembrolizumab monotherapy was initiated. Four days later, the patient experienced a persistent cough. Chest computed tomography showed that ground-glass opacity appeared on the same side as pleurodesis and spread bilaterally thereafter, which was diagnostic of immune checkpoint inhibitors (ICI)-related pneumonitis. As he presented a severe respiratory failure, corticosteroid therapy was administered. Two weeks later, respiratory failure completely resolved and the abnormal shadows dramatically improved. Our results indicate that severe ICI-related pneumonitis can develop within a short period after pleurodesis.

## Introduction

Pembrolizumab, an anti-programmed death ligand-1 (PD-1) monoclonal antibody, is widely used to treat lung cancer [1,2]. However, immune checkpoint inhibitors (ICIs) such as pembrolizumab are associated with immune-related adverse events (irAEs) including interstitial pneumonitis, which is potentially a fatal adverse event [3,4].

Malignant pleural effusion is sometimes observed in lung cancer patients at the first visit with an incidence of 26% [5]. Although pleurodesis is recommended for managing malignant pleural effusions, the safety of ICI treatment after pleurodesis is still unknown. There is only one case report of severe ICI-related pneumonia after talc pleurodesis. Here, we report a case of a lung adenocarcinoma patient with malignant pleural effusion who developed severe ICI-related pneumonitis after minocycline and OK-432 (picibanil) pleurodesis.

## Case presentation

**Figure 1:**
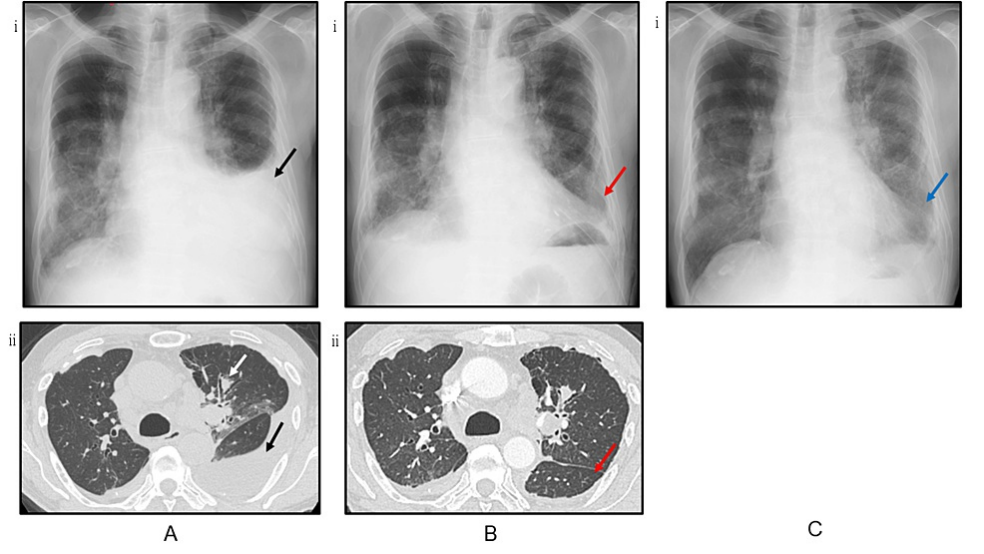
Radiological course before pembrolizumab administration. A. Chest X-ray (ⅰ) and CT (ⅱ) at first visit showed a nodule in the left upper lobe (white arrow, image ii) with bilateral pleural plaque. Left lung pleural effusion was also observed (black arrow, image i). B. After drainage, chest X-ray (ⅰ) and CT (ⅱ) showed that the left lung pleural effusion had disappeared (red arrows in images i and ii). C. Chest X-ray 12 days after pleurodesis showed a slight ground-glass opacity on the same side as pleurodesis (blue arrow).

A 74-year-old male patient with a smoking history visited our hospital due to dyspnea and a left pleural effusion on a chest X-ray by his previous doctor. He had no pre-existing medical conditions but he had a history of asbestos exposure because he had worked as an interior decorator. Because the pleural effusion was increasing, we performed chest drainage. Chest computed tomography (CT) showed a nodular shadow in the left upper lobe and bilateral mediastinal and hilar lymphadenopathy (Figures 1A, 1B). The specimen from pleural effusion revealed adenocarcinoma. After systemic examination, he was finally diagnosed with stage ⅣB (T4N3M1c) lung adenocarcinoma. The Oncomine Dx Target Test (Thermo Fisher Scientific, Inc, Tokyo, Japan) showed neither epidermal growth factor receptor (EGFR) mutations nor other driver mutations whereas the tumor cells strongly expressed PD-L1 (immunohistochemistry 22C3) with the tumor proportion score (TPS) of 95%. The patient received a single intrathoracic administration of minocycline at a dose of 100 mg and OK-432 5KE as pleurodesis after chest drainage. Although slight fever developed on the day of administration, no other complications were observed (Figure 1C).

**Figure 2:**
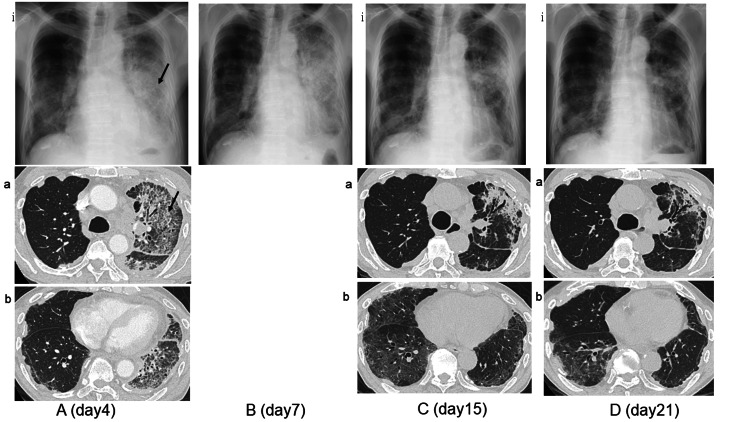
Radiological course after pembrolizumab administration. A. On day 4 after pembrolizumab administration, chest X-ray (ⅰ) and chest CT (a, b) revealed a widespread consolidation and ground-glass opacity on the same side as pleurodesis (black arrows). B. On day 7, the lung shadows apparently worsened. C. On day 15, the left lung shadow was dramatically improved whereas chest X-ray (ⅰ) and chest CT (a, b) showed the emergence of ground-glass opacity (GGO) in the right lower lobe. D. On day 21, chest X-ray (ⅰ) and chest CT (a, b) showed the bilateral shadows improved.

Based on tumor proportion score (TPS) high results, pembrolizumab monotherapy (200 mg/body) was administered 19 days after pleurodesis. Four days after pembrolizumab administration, the patient experienced a persistent cough, and chest CT revealed consolidation and ground-glass opacity (GGO) on the same side as pleurodesis (Figure 2A). Laboratory examinations showed an elevated C-reactive protein (CRP) level (14.02 mg/dL) and therefore, garenoxacin was initiated for consideration of bacterial pneumonia. However, oxygenation decreased to the point that O_2_ 5L/min at rest was required on day 7, and the left lung shadows apparently worsened (Figure 2B). Because laboratory examination showed elevated levels of serum KL-6 to 1434 U/L with negative serum procalcitonin and pneumonia appeared within a short period after pembrolizumab administration, the patient was considered to develop ICI-related pneumonitis (Common Terminology Criteria for Adverse Events (CTCAE) ver 5.0, Grade 3). We initiated intravenous methylprednisolone at a dose of 250 mg/day and then switched to oral prednisolone at a dose of 1mg/kg. Prednisolone was tapered off over six months. The left lung shadow dramatically improved on day 15 whereas chest CT showed the emergence of GGO in the right lower lobe (Figure 2C). On day 21, after the first administration of chemotherapy, oxygen therapy was terminated and the bilateral shadows apparently improved (Figure 2D).

## Discussion

In this case, OK-432 was administered for pleurodesis because OK-432 demonstrated comparable efficacy and safety profiles to talc-s in patients with lung adenocarcinoma [6]. Pembrolizumab was administered 19 days after pleurodesis using minocycline and OK-432. Chest CT showed that pneumonia developed limited on the same side as pleurodesis at first and spread bilaterally thereafter. Therefore, he was diagnosed as developing ICI-related pneumonitis. The clinical courses of the present case provide the following two clinical implications.

First, pleurodesis for malignant pleural effusion may facilitate the development of severe ICI-related pneumonitis. In fact, our patient developed severe ICI-related pneumonitis with respiratory failure after pleurodesis. Yokoe et al. reported interstitial pneumonitis associated with drugs such as EGFR-tyrosine kinase inhibitor and vinorelbine after pleurodesis [7]. This interstitial pneumonitis occurred bilaterally or on the side where pleurodesis was performed. However, it remains unclear whether ICI treatment following pleurodesis also leads to pneumonitis. To the best of our knowledge, there is only a case report about ICI-related pneumonitis following pleurodesis with talc slurry [8]. In this report, a 70-year-old male was treated with nivolumab 2 weeks after pleurodesis and subsequently developed a fatal bilateral interstitial pneumonitis. Taking this report together with the present case, it is important to recognize that severe ICI-related pneumonitis can occur after pleurodesis although other differentials such as acute lung injury after pleurodesis should be entertained.

Second, close follow-up is necessary for patients treated with ICI after pleurodesis. In this case, pneumonia appeared 4 days after pembrolizumab administration. Similarly, in the above-mentioned report of ICI-related pneumonitis after talc slurry pleurodesis, fever appeared on day 8 and pneumonia was diagnosed on day 12 [8]. On the other hand, an analysis by Nishino et al. showed that ICI-related pneumonitis developed 2.6 months in the median (range, 0.5-11.5) after initiating ICI although this paper did not describe whether pleurodesis was performed or not [9]. In addition, another paper reported that the median time to onset of ICI-related pneumonitis was 2.8 months (range, 9 days-19.2 months) [10]. Therefore, the time onset of ICI-related pneumonitis following pleurodesis in our case and the previous report [8] seems to be much shorter (4 and 12 days). Despite the unknown mechanism of the early onset of pneumonitis observed in the present case, pleurodesis is thought to result in the accumulation of cytokines in the pleural effusion and the activation of fibroblasts through the damage of pleural mesothelial cells [11]. These conditions may lead to the early onset of ICI-related pneumonitis. The clinical course of our patient, in which pneumonitis developed more severely on the same side as pleurodesis may support our hypothesis.

## Conclusions

We report a case of severe ICI-related pneumonitis developing in a short period after minocycline and OK-432 pleurodesis. Pleurodesis for malignant pleural effusion may facilitate the development of severe ICI-related pneumonitis. Chest physicians should be aware that close follow-up is important when using ICI after pleurodesis.
